# Editorial: Unraveling the influence of perivascular adipose tissue on vascular health

**DOI:** 10.3389/fphys.2026.1845969

**Published:** 2026-04-29

**Authors:** Maria A Delbin, Stephanie W Watts, Eduardo Nava

**Affiliations:** 1 Departamento de Biologia Estrutural e Funcional, Instituto de Biologia, Universidade Estadual de Campinas (UNICAMP), Campinas, São Paulo, Brazil; 2 Department of Pharmacology and Toxicology, College of Osteopathic Medicine, Michigan State University, East Lansing, MI, United States; 3 Department of Medical Sciences, University of Castile - La Mancha School of Medicine, Albacete, Spain

**Keywords:** chronic kidney disease, coronary artery bypass surgery (CABG), coronary artery disease, epicardial adipose tissue (EAT), PVAT (perivascular adipose tissue), sympathetic nerves, tissue clearing, vascular inflammation

## Introduction

Perivascular adipose tissue (PVAT) constitutes a critical interface in vascular regulation. Its role in modulating endothelium cells and the complexity of this crosstalk have sparked significant scientific interest, particularly regarding cardiovascular disease. Although the control of vascular function has been classically attributed to the endothelium, the overlapping roles of PVAT have arised with such a strenght that a further Research Topic to that published some years ago (Watts and Gollasch, 2018) was needed. Thus, we prepared this new Reseach Topic, entitled *Unraveling the Influence of Perivascular Adipose Tissue on Vascular Health*.

The key to the recognition of PVAT and the so called, *tunica adiposa* (Chaldakov et al., 2012), actually came from pharmacological functional studies rather than from histologists’, who otherwise had known for ages of the existence of fat around most vessels. It was very tempting to think that adipocytes close to the vessel acted as paracrine cells to the vessel wall by releasing transferable substances with vasomotor abilities. In this sense, adipocytes would operate in conjunction with intimal endothelial cells. Now we know that PVAT releases a variety of molecules that exceed in number those produced by endothelial cells. Both vascular layers — the vascular endothelium and PVAT — together release a vast amount of humoral signals toward the adjoining smooth muscle cells and the circulating blood. The interplay between endothelial and PVAT cells is extremely complex, especially considering the inflammatory phenotype PVAT adopts upon energy imbalance. Both a compensatory action of PVAT over endothelial dysfunction and an active influence of vascular disease and the subsequent endotheliopathy over the adjacent PVAT and its phenotypic profile have been proposed (Gil-Ortega et al., 2015).

In recent years, the role of PVAT in regulating vascular function has been increasingly recognized. PVAT influences vascular biology through multiple signals. It contains a variety of cell types, including adipocytes, endothelial cells, immune cells, and several other cell types. Bioactive molecules released from PVAT help regulate vascular homeostasis. The interaction between PVAT and the vessel wall makes this tissue a potential therapeutic target for cardiovascular disease. A deeper understanding of the biology of the interaction between the adipose tissue and the cardiovascular system is needed to identify new therapeutic targets and develop novel diagnostic biomarkers in cardiovascular medicine.

This Frontiers Research Topic is dedicated to highlighting both recognized and newly discovered functions of PVAT and the role it plays a role in both health and disease. The topic we present consists of two original works (Jackson et al. and Feng et al), two methodological papers (Watts et al. and Thompson et al.), three reviews (Hara and Sata; Sowa et al.; Dashwood et al.) and a perspective paper (Upadhaya et al.).

Jackson et al. investigate the sympathetic innervation of mesenteric PVAT in Sprague-Dawley and Dahl-salt sensitive (Dahl-SS) rats. The study utilizes sophisticated imaging techniques and immunofluorescent labeling of tyrosine hydroxylase to visualize sympathetic nerve fibers and CD-31 to identify the blood vessels perfusing the mesenteric PVAT. Tyrosine hydroxylase-positive structures were sparsely distributed within the mesenteric PVAT of both strains. Most identified sympathetic nerves were associated with small arteries and arterioles that support PVAT microcirculation suggesting limited direct sympathetic control over individual adipocytes. These findings are consistent in both male and female rats in the Dahl-SS model, raising the question that most adipocytes of rat mesenteric PVAT are regulated by distinct mechanisms.

The study carried out by Watts et al. demonstrates that rat thoracic aortic PVAT is vascularized. The authors provide a detailed methodological article for tissue preparation, clearing, visualizing and image analysis. The results validated the technique of this new protocol, supported the applicability with different rat vascular beds, revealed a dense presence of microvasculature in the ventral and lateral lobes of aortic PVAT, providing foundation for future studies focusing on microvascular density in health and disease.

The next methodological study is performed by Thompson et al. They propose a method to optimize and validate nuclei isolation from white adipose tissue in rats and cows, important animal models for studying cardiovascular and metabolic diseases. Their findings reveal limitations in nuclei isolation in rat white adipose tissue, mesenteric PVAT and retroperitoneal, which exhibit lower gene expression per cell compared to cow white adipose tissue. This highlights the challenges of single-nuclei RNA sequencing, which vary significantly depending on the species and the anatomical location of adipose tissue.

Emphasizing the importance of PVAT in vascular homeostasis, Hara and Sata review the role of PVAT and epicardial adipose tissue (EAT) in the pathogenesis of atherosclerosis. The authors first clarify the terminological definitions of these tissues and examine the relationship between PVAT inflammation and coronary artery disease, specifically focusing on the vascular wall at each stage of atherosclerotic development. They also report clinical evidence suggesting that increased epicardial adipose tissue volume is crucial in coronary atherosclerosis and may be used as a prognosis factor. Finally, potential therapeutic targets are discussed, including aerobic exercise, statin, SGLT2 inhibitor, GLP-1 receptor agonist and anti-inflammatory agent.

In a similar line of Hara and Sata‘s minireview, but focused on chronic kidney disease (CKD), Feng et al. demonstrate that EAT volume represents a predictive factor, in this case in non-dialysis CKD patients. Feng et al. carry out a prospective cohort study in more than 200 patients analysing the occurrence of cardiovascular outcomes (cardiovascular-related deaths, coronary revascularized patients and heart failure patients) of non-dialysis CKD patients and two computed tomography (CT) image parameters (adipose tissue volume and voxel average attenuation) of various thoracic PVAT depots, namely, peri-coronary adipose tissue (PCAT), thoracic peri-aortic adipose tissue (TAT), and EAT. It is shown that cardiovascular event development correlated with volumes obtained in these depots, particularly EAT, thus suggesting that fat volume as assessed by CT scaning can be used as a prediction tool in non-dialysis CKD patients.

A space in which PVAT may prove to be most immediately tractable clinically is in inflammation. Sowa et al. review the fat attenuation index (FAI) that can be used to assess the state of vascular inflammation. This has so far focused on the coronary arteries (as also reported by Hara and Sata) and aorta. While describing PVAT depots, molecular characteristics, and ‘beiging’, they raise an important question that is of absolute concern to this field: can PVAT be targeted therapeutically?

Drilling more specifically into EAT, Upadhaya et al. emphasize the clinical utility of imaging PVAT to understand how inflammation drives atherosclerosis. Coronary computerized tomography angiography (CCTA) is the method that allows for evaluating localized PVAT inflammation, with that specific to pericoronary adipose tissue attenuation termed PCATa. They delve into how this measure relates to obesity.

While most of the research carried out on PVAT deals with the medical benefits of this tissue in healthy conditions, Dashwood et al., focuses on the surgical benefits of PVAT. Indeed, PVAT displays its most practical side when it comes to protect bypass grafting and prevent vasospasm of arteries or veins used for coronary bypassing. In their review, Dashwood et al., explore the various grafting possibilities both with or without PVAT as well as the role of different PVAT-derived substances involved in the success of the bypasses, including prostaglandins, of which the first report on the existence of PVAT-derived prostaglandins is diputed (Ozen et al., 2013; Mendizábal et al., 2014).

Together, these basic and clinical studies advance our understanding of PVAT and further highlight the importance of EAT. Unraveling the fundamental mechanisms of PVAT structure and function is essential for vascular health; however, much remains to be explored. This Research Topic translates the findings into practical applications and inspires new fields of investigation.

## References

[B1] Chaldakov G. N. Beltowsky J. Ghenev P. I. Fiore M. Panayotov P. Rancic G. . (2012). Adipoparacrinology–vascular periadventitial adipose tissue (tunica adiposa) as an example. Cell Biol. Int. 36, 327–330. doi: 10.1042/CBI20110422, PMID: 22150107

[B2] Gil-Ortega M. Somoza B. Huang Y. Gollasch M. Fernandez-Alfonso M. S. (2015). Regional differences in perivascular adipose tissue impacting vascular homeostasis. Trends Endocrinol. Metab. 26, 367–375. doi: 10.1016/j.tem.2015.04.003, PMID: 26008879

[B3] Mendizábal Y. Llorens S. Nava E. (2014). Corrigendum to “Vasoactive effects of prostaglandins from the perivascular fat of mesenteric resistance arteries in WKY and SHROB. Life Sci. 100, 152. doi: 10.1016/j.lfs.2014.02.017, PMID: 33557003

[B4] Ozen G. Topal G. Gomez I. Ghorreshi A. Boukais K. Benyahia C. . (2013). Control of human vascular tone by prostanoids derived from perivascular adipose tissue. Prostaglandins Other Lipid Mediat. 107, 13–17. doi: 10.1016/j.prostaglandins.2013.06.002, PMID: 23791663

[B5] Watts S. W. Gollasch M. (2018). Editorial: perivascular adipose tissue (PVAT) in health and disease. Front. Physiol. 9. doi: 10.3389/fphys.2018.01004, PMID: 30104983 PMC6078060

